# The *mxd* operon in *Shewanella oneidensis* MR-1 is induced in response to starvation and regulated by ArcS/ArcA and BarA/UvrY

**DOI:** 10.1186/1471-2180-13-119

**Published:** 2013-05-27

**Authors:** Jana Müller, Soni Shukla, Kathinka A Jost, Alfred M Spormann

**Affiliations:** 1 Department of Civil & Environmental Engineering, Stanford University, Stanford, CA 94035-4020, USA; 2 Department of Chemical Engineering, Stanford University, Stanford, CA 94035-5025, USA; 3 Biomedical Engineering and Science, Stanford University, Stanford, CA 94035-5429, USA

## Abstract

**Background:**

*S. oneidensis* MR-1 is a dissimilatory metal-reducing bacterium. Under anoxic conditions *S. oneidensis* MR-1 attaches to and uses insoluble minerals such as Fe(III) and Mn(IV) oxides as electron acceptors. In the laboratory, *S. oneidensis* MR-1 forms biofilms under hydrodynamic flow conditions on a borosilicate glass surface; formation of biofilms was previously found to be dependent on the *mxd* gene cluster (*mxdABCD*).

**Results:**

This study revealed environmental and genetic factors regulating expression of the *mxd* genes in *S. oneidensis* MR-1. Physiological experiments conducted with a *S. oneidensis* MR-1 strain carrying a transcriptional *lacZ* fusion to the *mxd* promoter identified electron donor starvation as a key factor inducing *mxd* gene expression. Tn*5* mutagenesis identified the ArcS/ArcA two-component signaling system as a repressor of *mxd* expression in *S. oneidensis* MR-1 under planktonic conditions. Biofilms of ∆*arcS* and ∆*arcA* strains carrying a transcriptional *gfp* -reporter fused to the *mxd* promoter revealed a reduced *mxd* expression, suggesting that ArcS/ArcA are necessary for activation of *mxd* expression under biofilm conditions. Biofilms of ∆*arcS* and ∆*arcA* mutants were unable to form a compact three-dimensional structure consistent with a low level of *mxd* expression. In addition, BarA/UvrY was identified as a major regulator of *mxd* expression under planktonic conditions. Interestingly, biofilms of ∆*barA* and ∆*uvrY* mutants were able to form three-dimensional structures that were, however, less compact compared to wild type biofilms.

**Conclusions:**

We have shown here that the *mxd* genes in *S. oneidensis* MR-1 are controlled transcriptionally in response to carbon starvation and by the ArcS/ArcA and the BarA/UvrY signaling system. BarA might function as a sensor to assess the metabolic state of the cell, including carbon starvation, leading to expression of the *mxd* operon and therefore control biofilm formation.

## Background

*Shewanella oneidensis* MR-1 is a dissimilatory metal-reducing bacterium [1] and can use under anoxic conditions insoluble Fe(III) and Mn(IV) oxide minerals as electron acceptors [2,3]. In the laboratory, *S. oneidensis* MR-1 forms biofilms under hydrodynamic flow conditions on a borosilicate glass surface, where biofilm formation is mediated by a set of complementary molecular machineries, comprised of the type IV MSHA pilus and a putative exopolysaccharide biosynthesis (EPS) gene cluster (*mxdABCD)*[4,5]. The first gene of this cluster is *mxdA*, which is predicted to encode for a gene with unknown function; however, MxdA was recently shown to control indirectly cellular levels of c-di-GMP in *S. oneidensis* MR-1 [6]. *MxdB* has homology to a membrane-bound type II glycosyl transferase and was thought to be involved in the transport of extracellular material involved in forming the matrix of *S. oneidensis* MR-1 biofilms. This hypothesis was supported by genetic analysis revealing that ∆*mxdB* mutants were unable to transition from a cell monolayer to a three dimensional biofilm structure [4]. *MxdC* shares homology with an efflux pump and *mxdD* was annotated as a conserved hypothetical protein with no known homology. ∆*mshA*∆*mxdB* double mutants were entirely deficient in initial attachment and biofilm formation [5]. Expression of adhesion factors such as EPS are regulated in *Vibrio cholerae, Escherichia coli* and *Pseudomonas aeruginosa* in response to environmental factors. The *vps* gene cluster in *V. cholerae*, for example, was shown to be controlled in a cell- density dependent manner [7-10] involving several two-component signaling systems (TCS).

The global regulator ArcA is part of the ArcS/ArcA two-component regulatory system in *S. oneidensis* MR-1 [11-14]. Recently, it was shown that phoshorylation of ArcA by ArcS requires the presence of HptA, a separate phosphotransfer domain [14]. HptA of *S. oneidensis* MR-1 shares homology with the N-terminal domain of ArcB, the sensor histidine kinase of the *E. coli* ArcB/ArcA system, but does not share significant homology with ArcS from *S. oneidensis* MR-1. ArcS/HptA have been shown to functionally complement an *E. coli* ΔArcB mutant [13]. In *E. coli*, ArcA is part of the ArcB/ArcA (anaerobic respiration control) two-component regulatory system, a major regulator of gene expression involved in aerobic/anaerobic respiration and fermentative metabolism [15-20]. A recent study investigated the domain structure of ArcS in *S. oneidensis* MR-1 and revealed significant differences when compared to *E. coli* ArcB [21]. It was shown that in the N-terminal part, ArcS possesses a CaChe-sensing domain, two cytoplasmic PAS-sensing and two receiver domains. Due to the expanded sensory region, ArcS of *Shewanella* species might be able to respond to a wider array of environmental signals and is not restricted to changing redox conditions.

ArcA has been previously shown to play a role in biofilm formation in *S. oneidensis* MR-1. *S. oneidensis* MR-1 ∆*arcA* mutants form biofilms with about 70% less biomass on a borosilicate glass surface under hydrodynamic flow conditions and are unable to mature into a highly three-dimensional biofilm structure when compared to wild type [22].

In this study, we investigated physiological and genetic factors involved in the regulation of the *mxd* operon in *S. oneidensis* MR-1. We found that *mxd* expression was induced by carbon starvation. The TCS ArcS/ArcA was discovered to constitute a major activator of the *mxd* genes under biofilm conditions, and to repress *mxd* expression under planktonic conditions. BarA/UvrY was identified as a major inducer of *mxd* expression under planktonic conditions and appeared to have a minor role in biofilm formation.

## Results

### ∆*mxdA* and ∆*mxdB* mutant cells are deficient in cell-cell aggregation when grown planktonically under minimal medium conditions

**Figure 1:**
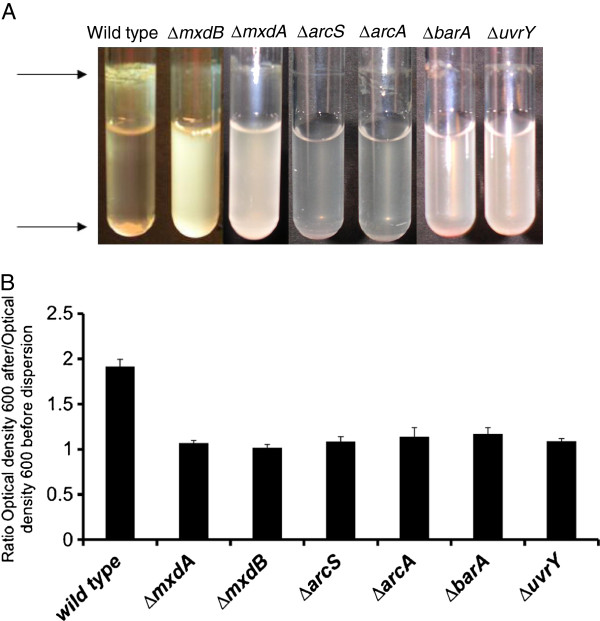
**Cell aggregation and biofilm formation of *S. oneidensis *MR-1 wild type and mutants.** (**A**) Cell aggregation and biofilm formation of *S. oneidensis* MR-1 wild type and mutants in planktonic culture under minimal medium conditions. See Materials and Methods for details. Note the thick biofilm ring in the wild type culture at the top (top arrow) and aggregated cells on the bottom of the tube (lower arrow). (**B**) Quantification of cell aggregation in *S. oneidensis* MR-1 wild type and mutants in planktonic culture under minimal medium conditions. The ratio of the optical density measured at 600 nm of wild type and mutant cultures after and before dispersion was used to determine their aggregation phenotypes.

Wild type *S. oneidensis* MR-1 cells, when grown for 16 h in a liquid minimal medium, formed a thick biofilm ring at the air-liquid interface on the borosilicate surface of a test tube (Figure 1A). Stationary phase cultures (OD_600_~ 3.2) aggregated in a rotating culture test tube and quickly settled to the bottom of the tube when rotation was arrested for 10 minutes (Figure 1A). We took advantage of this aggregation phenotype and developed a quantitative aggregation assay by calculating the ratio of the optical density, measured at 600 nm, of cells before and after dispersion by rigorously vortexing (Figure 1B). Analyzing wild type and mutants by this assay, we found ∆*mxdA* and ∆*mxdB* mutant cultures to be deficient in aggregation (Figure 1). Consistent with this observation, the biomass of biofilms of these strains that formed at the air-liquid interface on the borosilicate glass test tube surface was dramatically reduced relative to wild type. Notably, the described aggregation and adhesion phenotypes were not observed under LB medium conditions.

These data indicate a possible role for *mxdA* and *mxdB* in cell-surface adhesion when growing in minimal medium. When comparing growth rates in LB to minimal medium, we found no correlation between growth rate and *mxd* expression, suggesting that a low growth rate, as found under starvation conditions in minimal medium, was most likely not responsible for *mxd* induction (data not shown).

**Figure 2:**
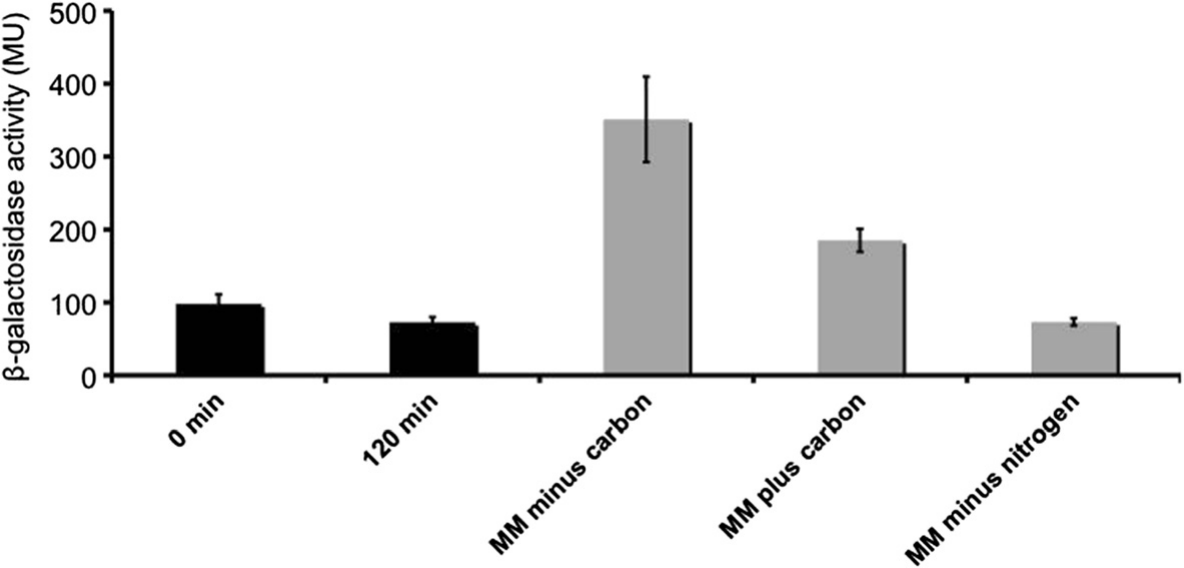
***Mxd *expression in *S. oneidensis *MR-1 wild type.***Mxd* expression in *S. oneidensis* MR-1 wild type cells exposed to carbon and nitrogen starvation conditions. Wild type cells carrying the *mxd* promoter transcriptionally fused to *lacZ* were grown under complex medium conditions to mid-exponential phase. Cells were pelleted and the supernatant was discarded. Subsequently, cells were either resuspended in minimal medium containing no carbon source (MM minus carbon, or in minimal medium amended with 50 mM sodium lactate (MM plus carbon), or in minimal medium with the nitrogen source omitted (MM minus nitrogen). A set of control samples (black bars) was pelleted and resuspended in the same medium. Samples were assayed for *β*-galactosidase activity. Data represent an average of three independent experiments.

We therefore hypothesized that limitation for essential nutrients or accumulation of metabolites might be involved in *mxd* induction, and specifically tested whether carbon or nitrogen limitation induced *mxd* expression. For this purpose we constructed a wild type *mxd*::*lacZ* reporter strain (AS832) (see Table 1 and 2). This strain was grown in LB medium to an OD_600_=0.3. Cells were pelleted, resuspended in minimal medium amended with 50 mM sodium lactate, incubated for 120 minutes at 30°C and subsequently assayed for specific *β*- galactosidase activity. Similarly, cells were also exposed to minimal medium without carbon or nitrogen source. As a control, cells were resuspended in the same LB culture medium. As shown in Figure 2 no increase in *mxd* expression was observed when cells were incubated in the LB culture medium for 120 minutes (Figure 2) and compared to the same sample at t=0 minutes. Similarly, cells exposed to minimal medium void of a nitrogen source also did not show any increase in *mxd* expression. Cells exposed to minimal medium supplemented with lactate led to minor *mxd* induction. However, shifting cells to minimal medium void of a carbon source led to significant *mxd* induction (~400 MU). Thus, starvation for carbon appears to be important for *mxd* expression in *S. oneidensis* MR-1.

**Table 1:** Strains used in this study

**Strain**	**Relevant genotype or description**	**Source or reference**
***E. coli***		
S17-lambda pir	*thi pro recA hsdR* [RP4-2Tc::Mu-Km::tn7]lambda pir Tp^r^ Sm^r^	[38]
AS259 (BW20767)	RP4-2-Tc::Mu-1 Kan::Tn7 integrant *leu-63*::IS10 *recA1 zbf-5 creB510 hsdR17 endA1 thi uidA (deltaMluI)::pir*^*+*^	[12]
AS262	S17-lambda pir harbouring pUX-BF13	[39]
AS392	S17-lambda pir harbouring pGP704-mini-Tn7(Gm) *P*_A1/04/03-GFPmut3*_	[39]
***S. oneidensis***		
AS93	*S. oneidensis* MR-1, wild type, tagged with GFPmut3* in a Tn7 construct, Gen^r^	[12]
AS536	AS93 harbouring pME6031(Tc)::P*mxd*^*-300+*1^*lacZ* (pJM1)	This study
AS556	AS93 harbouring pME6031(Tc)::*lacZ* (promoterless)	This study
AS579 (MR-1)	*S. oneidensis* MR-1, wild type	PNNL
AS829	In-frame deletion of *mxdA* in MR-1	This study
AS830	MR-1 tagged with GFPmut3* in a Tn7 construct, Gen^r^	This study
AS831	In-frame deletion of *mxdB* in MR-1 tagged with GFPmut3* in a Tn7 construct, Gen^r^	This study
AS832	MR-1 harbouring pME6031(Tc)::P*mxd*^*-300+1*^*lacZ*	This study
AS833	MR-1 harbouring pME6031(Tc)::P*mxd*^*-150+1*^*lacZ*	This study
AS834	MR-1 harbouring pME6031(Tc)::P*mxd*^*-100+1*^*lacZ*	This study
AS835	MR-1 harbouring pME6031(Tc)::*Pmxd*^*0+1*^*lacZ*	This study
AS837	MR-1 harbouring pProbe-NT(Kan)::P*mxd*^*-300+1*^*gfp*	This study
AS838	MR-1 harbouring pProbe-NT(Kan):: *gfp (*promoterless)	This study
AS839	In-frame deletion of *arcA* in MR-1	This study
AS840	AS839 tagged with GFPmut3* in a Tn7 construct, Gen^r^	This study
AS841	In-frame deletion of *arcS* in MR-1	This study
AS842	AS841 tagged with GFPmut3* in a Tn7 construct, Gen^r^	This study
AS843	In-frame deletion of *uvrY* in MR-1	This study
AS844	AS843 tagged with GFPmut3* in a Tn7 construct, Gen^r^	This study
AS845	In-frame deletion of *barA* in MR-1	This study
AS846	AS845 tagged with GFPmut3* in a Tn7 construct, Gen^r^	This study
AS855	AS839 harbouring pProbe-NT(Kan)::P_*mxd*_^*-300+1*^*gfp (*pJM6)	This study
AS856	AS841 harbouring pProbe-NT(Kan)::P_*mxd*_^*-300+1*^*gfp* (pJM6)	This study
AS860	AS841 harbouring pME6031(Tc)::P_*mxd*_^*-300+1*^*lacZ*	This study
AS861	AS845 harbouring pME6031(Tc)::P_*mxd*_^*-300+1*^*lacZ*	This study
AS862	AS843 harbouring pME6031(Tc)::P_*mxd*_^*-300+1*^*lacZ*	This study
AS863	AS839 harbouring pME6031(Tc)::P_*mxd*_^*-300+1*^*lacZ*	This study
AS864	AS847 harbouring pME6031(Tc)::P_*mxd*_^*-300+1*^*lacZ*	This study
AS865	MR-1 harbouring pME6031(Tc)::*lacZ* (promoterless)	This study

**Table 2:** Primers used in this study

**Primer**	**Sequence (5’-3’)**
Pmxd-fw-SphI	TCTTGGCATGCCATTATTAAATGACC
Pmxd-rv-XbaI	TGTCATCTAGAAAACCTTGTACAGAT
LacZ-fw-XbaI	GGAATCTAGAATGACCATGATTACGGATT
LacZ-rv-PstI	AGAACTGCAGGCAAAAATAATACCCGTATC
Pmxd-fw-150-SphI	CGTACTACGCATGCGATTAAAGCGAGTTTTGATATTC
Pmxd-fw-250-SphI	CGTACTACGCATGCTTATTTTTATTTTGTTATTTTTTAATATTC
Pmxd-fw-300-SphI	CGTACTACGCATGCTTCAGGGTGAACAATTGTATC
Pmxd-fw-HindIII	GTACGTCAAAGCTTCATTATTAAATGACC
Pmxd-rv-XbaI	TGTCATCTAGAAAACCTTGTACAGAT
SO1860_uvrY_F-O	GCGAGCTAGAATAGGGCTAGGT
SO1860_uvrY_F-O	TCTTGCTGCTCTGCCACTC
SO1860_uvrY_5-O	CTCATTCTAAGCGCAGCTCCT
SO1860_uvrY_5-I	AACCCGTCGGAGGGATAATTGCATAACTGGCATATTCATGTCC
SO1860_uvrY_3-I	CAATTATCCCTCCGACGGGTTGAGAATCCGTTTAAAGCCTTATCTG
SO1860_uvrY_3-O	ACTGCAGCGGGATAACTGGTA
SO3457_barA_F-O	TTTATCATCGAGCAATTGGTAAACA
SO3457_barA_R-O	GAGTTCAAGGTAATAATTGACTAAACG
SO3457_barA_5-O	GTCGGCTGCAATCAACTCTAA
SO3457_barA_5-I	ATCTATACGCTCGCGGTGTTCATGTTGTTGACAGGGTTCATA
SO3457_barA_3-I	ACACCGCGAGCGTATAGATCTTTAAGCGTTTAAAAGCGCTAGAACTACCACA
SO3457_barA_3-O	CGCTGTTTGTTAAGATAAATCCTTG
SO3988_arcA_I_rv	AGTTACCACATACCCTTCTGCCTCG
SO3988_arcA_I_fw	TGAGTCATGTTGTCCATCGGTAGTC
SO3988_arcA_check_rv	GCGTTGCAGGACGAAGGCAAGTTG
SO3988_arcA_check_fw	CAACGGCGTTTGATAATGCTGCCAC
SO3988_arcA_II_rv	TCTAAGCATTCAATGCGTGG
SO3988_arcA_II_fw	GTGACTATCCGTCGTATCCGTAAGC

### ArcS/ArcA functions as a repressor of the *mxd* operon in planktonic cells

**Figure 3:**
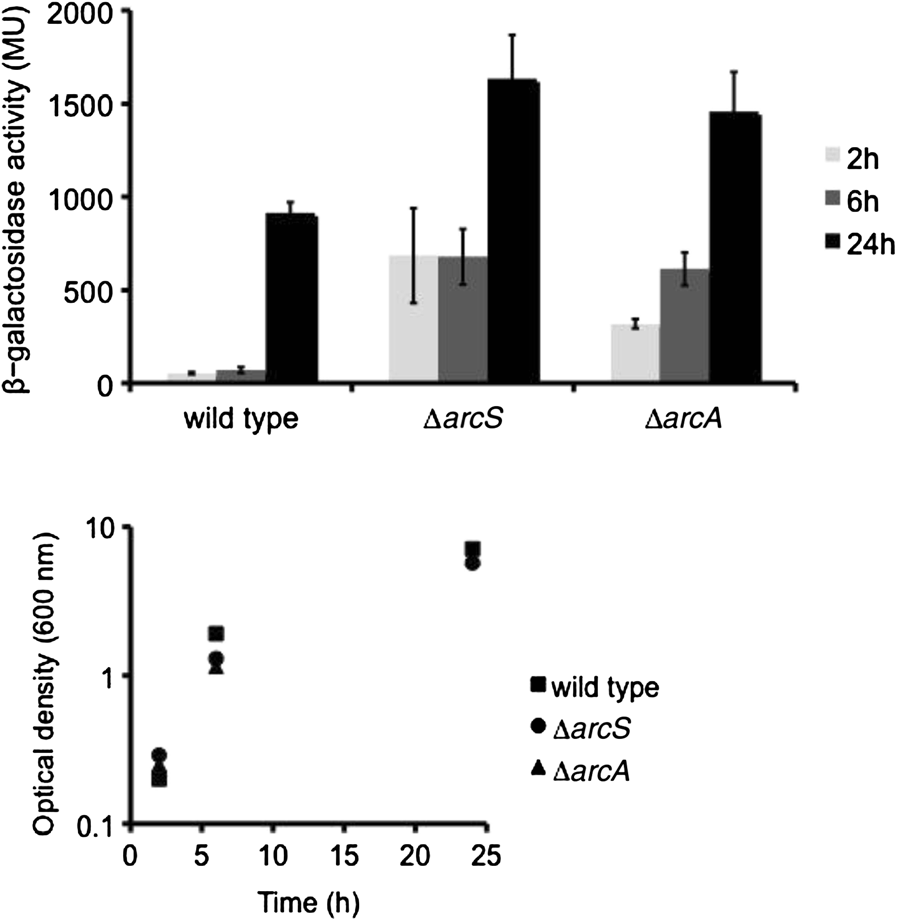
***Mxd *expression in *S. oneidensis *MR-1 wild type, ∆*arcS *and ∆*arcA *mutants.***Mxd* expression in *S. oneidensis* MR-1 wild type, ∆*arcS* and ∆*arcA* mutant cells grown under LB medium conditions. Wild type, ∆*arcS* and ∆*arcA* mutants carrying the *mxd* promoter transcriptionally fused to *lacZ* were grown under LB medium conditions for 24 h. Cells were harvested after 2 h, 6 h or 24 h and assayed for *β*-galactosidase activity. Optical densities are shown for all time points. Data represent an average of three independent experiments.

Tn*5* mutagenesis was performed to identify genes regulating *mxd* expression. We subjected the wild type *mxd*::

*lacZ* reporter strain (AS832) to four independent rounds of Tn*5* transposon mutagenesis. A total of 12,000 Tn*5* insertion mutants were qualitatively screened for deregulated *mxd* expression by visually comparing colours of Kan-resistant colonies plated on X-gal plates relative to the parental strain. 48 out of 12,000 Tn*5* insertion mutants were identified either as a loss- or gain-of-function mutants, respectively. After quantitative confirmation of the Tn*5* mutant phenotypes by *β*-galactosidase assays (data not shown), Tn*5* insertion sites were mapped. Among the selected Tn*5* mutants, we found in two independent mutageneses insertions in the response regulator ArcA and its cognate histidine sensor kinase ArcS associated with a gain-of-function phenotype. In order to exclude polar effects due to the Tn*5* insertions, we constructed in a wild type background marker-less in-frame deletions of *arcS* (AS841) and *arcA* (AS839), respectively (see Table 1 and 2). We then introduced the *mxd*::*lacZ* construct into these strains to generate strains AS860 and AS863, respectively, and examined *mxd* expression in these mutants when grown under LB medium conditions. As data in Figure 3 (top) show, a 12 times higher *mxd* expression in exponentially growing cells and about 1.5 times higher *mxd* expression in stationary phase cells was observed relative to wild type. Our data show that ArcS/ArcA is a major transcriptional repressor of *mxd* under planktonic conditions, and represses the *mxd* operon primarily in exponentially growing cells.

**Figure 4:**
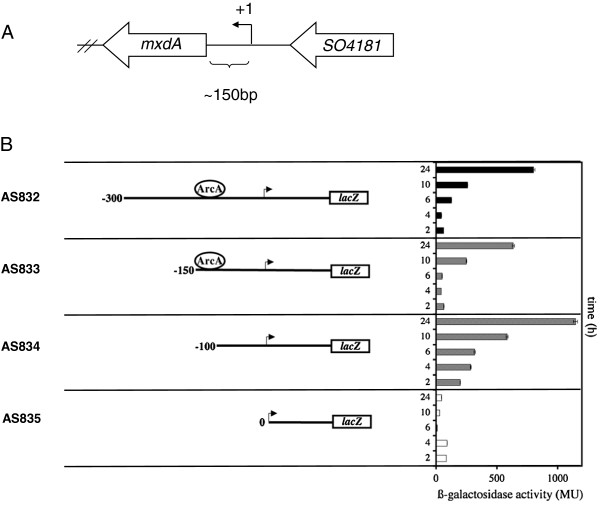
**Characterization of the *mxd *promoter.** (**A**) Schematic representation of the *mxd* transcription start site (+1). (**B**) Wild type strains carrying reporter constructs with truncated *mxdA* up-stream regions transcriptionally fused to *lacZ* were grown under complex media conditions. The different strains were assayed for *β*-galactosidase activity, expressed as Miller Units (MU). The cartoon on the left side shows a graphical representation of the truncated *P*_*mxd*_*::lacZ* constructs. The construct marked 0 contains a fragment corresponding to 150 bp upstream of the *mxdA* translation initiation site, representing the approximate transcription start site. The constructs marked -100, -150 and -300 contain fragments corresponding to 100, 150 and 300 bp upstream of the approximate transcription start site and correspond to strains AS834, AS833 and AS832. The graph on the right side shows the corresponding *β*-galactosidase activities (y-axis) for cells harvested after 2 h, 4 h, 6 h, 10 h and 24 h (x-axis). A predicted ArcA binding site at position -112 bp is indicated.

Further support for a direct role of the ArcS/ArcA system in control of *mxd* expression comes from a *mxd* promoter deletion analysis. The *mxd* transcription start site (+1) was experimentally determined by primer extension analysis and mapped at -150 bp (data not shown and Figure 4A). *In silico* analysis of the *mxd* promoter predicted putative ArcA binding sites at -29 bp, -86 bp and -112 bp upstream of the *mxd* transcription start site (Figure 4A and B). In order to experimentally test these predictions, we created truncations in the putative *mxd* promoter region, and transcriptionally fused the truncated promoters to *lacZ,* yielding strains AS832-835 (Figure 4B) (see Table 1 and 2). All strains were grown in LB medium, and cells from early exponential phase (2 h) through late stationary phase (24 h) were harvested

 and assayed for *β*-galactosidase activity (Figure 4B). Interestingly, when deleting the region upstream of -100 bp from the transcriptional start site (AS834), expression was increased about eightfold during exponential growth phase (> 6 h) compared to reporter strains carrying *mxd* upstream regions deleted to -150 bp (AS833) and -300 bp (AS832) (Figure 4B). As the ArcA binding sites were predicted at -29 bp, -86 bp and -112 bp upstream of the *mxd* transcriptional start site, the predicted -112 bp ArcA binding site is deleted in the -100 bp reporter strain (AS834), thus abolishing putative ArcA binding. Collectively, the observed data are consistent with the hypothesis that ArcS/ArcA is a major transcriptional repressor of the *mxd* operon under planktonic conditions.

### BarA/UvrY is a major activator of *mxd* expression in planktonic cells

**Figure 5:**
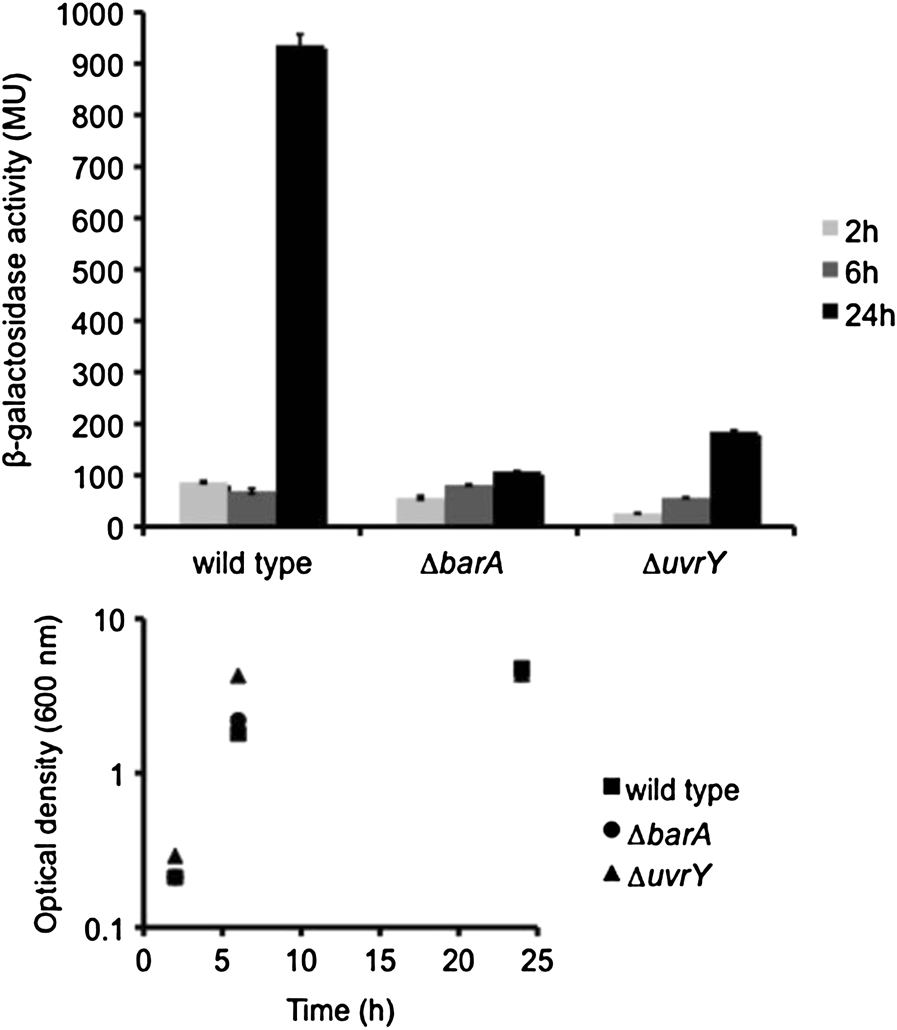
***Mxd *expression in *S. oneidensis *MR-1 wild type, ∆*barA *and ∆*uvrY *mutants.***Mxd* expression in *S. oneidensis* MR-1 wild type, ∆*barA* and ∆*uvrY* mutant cells grown under LB medium conditions. Wild type, ∆*barA* and ∆*uvrY* mutants carrying the *mxd* promoter transcriptionally fused to *lacZ* were grown under LB medium conditions for 24 h. Cells were harvested after 2 h, 6 h or 24 h and assayed for *β*-galactosidase activity. Optical densities are shown for all time points. Data represent an average of three independent experiments.

In the above reported transposon mutageneses, we also identified *uvrY* (SO1860) to transcriptionally control *mxd*. Recently biochemical evidence showed that BarA

 is the cognate sensor histidine kinase of UvrY, and that BarA/UvrY in *S. oneidensis* MR-1 constitute a functional two-component regulatory system [23]*.* We therefore constructed markerless in-frame deletions of *barA* (AS845) and *uvrY* (AS843), inserted the *mxd*::*lacZ* construct into each strain, and determined *mxd* expression in strains AS861 (AS845 background) and AS862 (AS843 background) under LB medium conditions (see Table 1 and 2). As evident from Figure 5 (top), both ∆*barA* and ∆*uvrY* mutants showed drastically reduced *mxd* expression primarily in stationary phase. Furthermore, we observed that ∆*barA* and ∆*uvrY* mutant strains, when grown for 24 h under minimal medium conditions, failed to aggregate under planktonic conditions, similar to a ∆*mxdB* (AS831) mutant (Figure 1A and Figure 5). These data provide genetic evidence that BarA/UvrY might function as an activator of the *mxd* operon under planktonic growth conditions. This conclusion is further supported by the observation that ∆*barA* and ∆*uvrY* mutants exhibit a ∆*mxdB* phenotype when grown planktonically in minimal medium.

### ArcS/ArcA and BarA/UvrY regulate formation of hydrodynamically-grown biofilms

**Figure 6:**
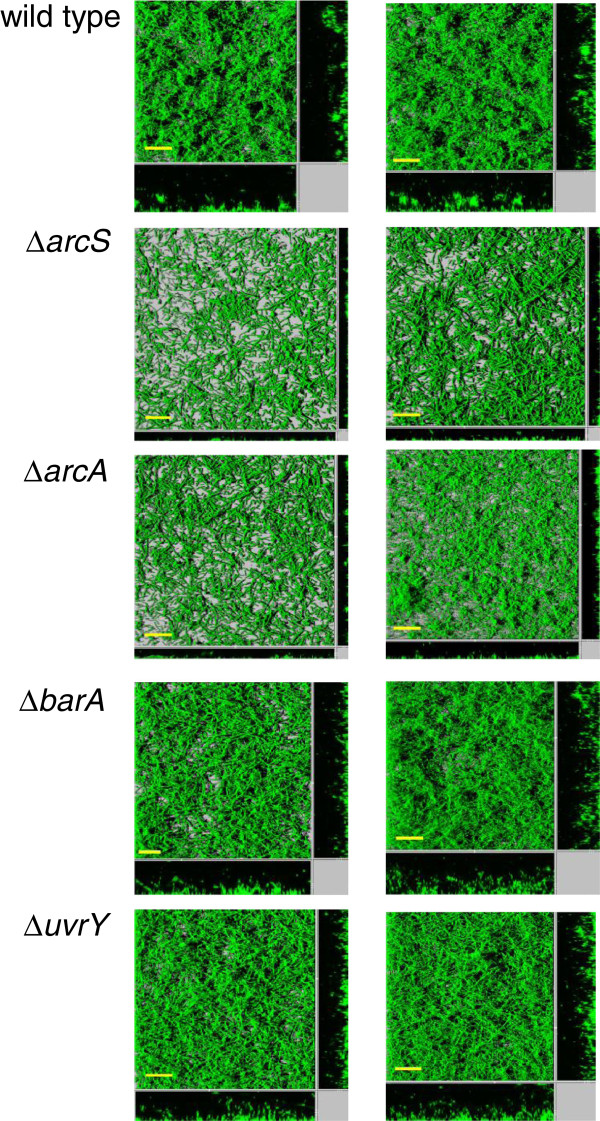
**Biofilms of *S. oneidensis *MR-1 wild type, ∆*arcS*, ∆*arcA*, ∆*barA *and ∆*uvrY *mutants.** CLSM images of *S. oneidensis* MR-1 wild type, ∆*arcS*, ∆*arcA*, ∆*barA* and ∆*uvrY* mutant biofilms grown in LM in a hydrodynamic flow chamber. CLSM images were taken at 24 h (left column) and 48 h (right column) post-inoculation. Scale bars are 30 μm.

The above data showed that ArcS and ArcA act as repressors of *mxd* expression, whereas BarA and UvrY strongly activate *mxd* expression under planktonic growth conditions. We next examined whether these regulators have a function under biofilm conditions. Biofilms of wild type, ∆*arcS*, and ∆*arcA* mutants were grown under hydrodynamic biofilm conditions, and biofilms were imaged by CLSM at 24 h and 48 h post-inoculation. Interestingly, both ∆*arcS* and ∆*arcA* mutant biofilms were unable to form a three-dimensional biofilm structure, and their biofilms were of similar structure as *mxd* mutant biofilms (Figure 6). As this finding was opposite to what we had expected based on the ∆*arcS* and ∆*arcA* mutant phenotypes in planktonic cells, we examined whether the biofilm phenotype of ∆*arcS* (AS842) and ∆*arcA* (AS840) mutants was indeed due to down-regulation of *mxd*. A transcriptional P_*mxd*_::*gfp* reporter strain was constructed and introduced into wild type (AS837), ∆*arcS* (AS856) and ∆*arcA* (AS855), respectively. Biofilms of wild type (AS837), ∆*arcS* (AS856) and ∆*arcA* (AS855) carrying the P_*mxd*_ ::*gfp* reporter were grown for 24 h in LM medium, harvested from the flow chamber and analyzed by flow cytometry for GFP fluorescence intensity (see Table 1 and 2). To account for non-specific background signals, a wild type strain carrying a promoterless *gfp* -reporter construct (AS838) was used as a control. While on average about 40% of the cells derived from a wild type biofilm showed P_*mxd*_-dependent GFP fluorescence above background, only about 1% of the cells from ∆*arcS* and ∆*arcA* biofilms did so (Additional file 1: Figure S1), consistent with the previously observed biofilm defect. These data provide evidence at the level of global biofilm structure as well as of single cell gene expression that ArcS/ArcA is necessary for significant positive activation of *mxd* expression in *S. oneidensis* MR-1.

∆*barA* and ∆*uvrY* mutants formed well-developed three-dimensional structures that were less compact compared to wild type (Figure 6). These data therefore suggest that BarA/UvrY plays only a minor regulatory role under biofilm conditions.

## Discussion

### Carbon starvation induces *mxd* gene expression in *S. oneidensis* MR-1

**Figure 7:**
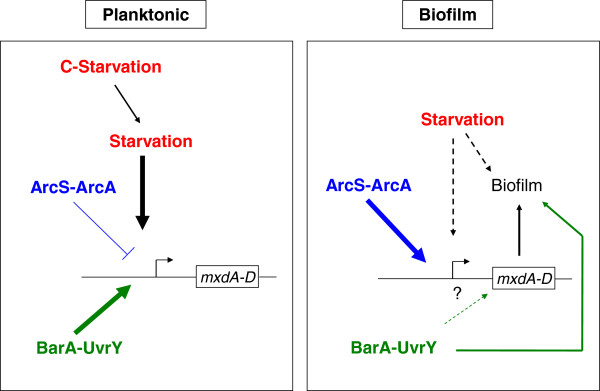
**Summary: *Mxd *regulation in *S. oneidensis *MR-1.** Summary of *mxd* regulation in *S. oneidensis* MR-1 under planktonic (left cartoon) and biofilm (right cartoon) conditions. Under planktonic conditions starvation and more specifically carbon starvation was identified to transcriptionally induce expression of the *mxd* operon. The ArcS/ArcA TCS was found to act as a minor repressor of the *mxd* genes under planktonic conditions. The TCS BarA/UvrY was identified to induce *mxd* gene expression under planktonic growth conditions. Under biofilm conditions, the ArcS/ArcA TCS activates *mxd* gene expression which is contrary to the findings under planktonic conditions. The TCS BarA/UvrY was found to act as a minor inducer of biofilm formation (solid arrow) and it remains to be determined if it acts via the *mxd* operon (dashed arrow).

While investigating physiological factors inducing *mxd* expression in *S. oneidensis* MR-1, we discovered that expression of the *mxd* genes in *S. oneidensis* MR-1 were regulated differentially depending on whether carbon starvation conditions prevailed under planktonic or biofilm conditions (Figure 7). The data showed furthermore that *arcA*/*arcS* as well as *barA*/*uvrY* are important regulators of *mxd* expression although under different conditions (Figure 7).

Consistent with our data, earlier findings in *P. aeruginosa* and *E. coli* had shown that nutrient-depletion

 enhanced biofilm formation, while high concentrations of nutrients repress the formation of biofilms [24,25]. In nature, accessible organic carbon is often scarce and can be found sorbed to surfaces such as organic-rich flocculates of marine snow and fecal pellets. Being able to sense and respond to changing carbon concentrations in these environments is crucial to the survival of bacteria. While starvation for carbon generally leads to a decrease in growth rate and metabolic activity in bacteria, our data suggest that *S. oneidensis* MR-1 cells activate production of adhesion factors responsible for biofilm formation under these conditions. This acclimation strategy could potentially confer an ecological advantage for *S. oneidensis* MR-1 cells interacting with a carbon rich surface, as they are already primed for adhesion.

### BarA/UvrY functions as an activator of the *mxd* genes under planktonic growth conditions and has a role in the regulation of biofilm formation

We showed here that BarA/UvrY activates *mxd* expression under organic rich medium conditions when planktonic cells entered stationary phase (Figure 7). BarA/UvrY is highly conserved in Gram-negative bacteria, and controls a variety of physiological functions including carbon storage [26-30]. In carbon storage regulation (Csr) BarA/UvrY regulates small RNAs controlling elements of this pathway, which are major posttranscriptional regulators of biofilm formation in *E. coli*[31]. The stimuli for the BarA sensor histidine kinase in *E. coli* are aliphatic carboxylic acids, such as formate, acetate, propionate and others, providing a physiological signal reflecting the metabolic state of cells and thereby linking posttranscriptional control by the Csr system with central metabolism [30].

Interestingly, *S. oneidensis* MR-1 biofilms of both ∆*barA* and ∆*uvrY* mutants formed less compact biofilms when grown under hydrodynamic flow conditions. Based on these data and the above discussed findings that low carbon concentration induces *mxd* expression, we hypothesize that BarA might function as a sensor for carbon starvation, e.g., at high cell density when nutrients become growth limiting in planktonic culture. We hypothesize that under these conditions starvation-sensing BarA signals to UvrY, which, in return, directly or indirectly activates *mxd* expression and, by this cascade, controls biofilm formation. Homologous of BarA/UvrY have been shown to control secondary metabolism, including the excretion of biofilm exopolysaccacharides in other γ-proteobacteria [32-36]. In the closely related bacterium *Pseudomonas fluorescens*

 production of several antibiotic-like secondary metabolites is regulated by the orthologs GacA/GacS and via the small RNAs RsmXYZ [37]. In *P. fluorescens* expression of these small RNAs was found to be positively controlled by GacS/GacA at high cell density and intermediates of central metabolism such as 2-oxoglutarate, succinate and fumarate which may be present at elevated intracellular concentration under conditions when cells are electron acceptor-limited [37]. It is conceivable that *S. oneidensis* MR-1*,* similar to *P. fluorescens*, senses its metabolic state at the level of primary metabolites, and uses the level to control aspects of secondary metabolism including biofilm formation. The BarA/UvrY system and its components have been studied to some extent in *S. oneidensis* MR-1 [23]. It was found to contain all major components of the BarA/UvrY/Csr pathway*.* UvrY in *S. oneidensis* MR-1 positively regulates the two small RNAs, *csrB1* and *csrB2* and a corresponding CsrA ortholog was also identified. The transcriptional fusion construct used in our study, which contains the *mxd* Shine Dalgarno sequence, is able to reflect control in *mxd* expression on the transcriptional and posttranscriptional level. Therefore, the decreased *mxd* expression detected in the *barA* and *uvrY* mutants might be a result of transcriptional regulation by *uvrY* which directly or indirectly interacts with the *mxd* promoter or a posttranscriptional control possibly via CsrA or both.

Interestingly, *S. oneidensis* MR-1 biofilms of ∆*barA* and ∆*uvrY* mutants were only partially defective (Figure 6). These biofilm defects might be a consequence of the idiosyncrasy of a biofilm environment: microbial biofilms are nutrient-stratified environments where cells at the surface of the biofilm have better access to nutrients, including oxygen, whereas cells in the layers distant from the planktonic interface become increasingly nutrient limited. If the BarA/UvrY system responds to lower concentrations of organic substrates, this regulator might be activated in the deeper, nutrient-deprived layers of the biofilm. Consequently, in the absence of BarA or UvrY part of the biofilm population would not express the *mxd* genes and confer adhesion, leading to a loosely structured biofilm such as observed in ∆*barA* and ∆*uvrY* mutants.

### The ArcS/ArcA TCS functions as a repressor of the *mxd* genes under planktonic growth conditions and activates the *mxd* operon in a biofilm

We identified and showed here that the ArcS/ArcA system controls *mxd* expression in *S. oneidensis* MR-1. Even though a role for ArcA in *S. oneidensis* MR-1 biofilm formation was previously introduced, no mechanistic explanation was provided. Our data show that ArcS/ArcA act as a repressor of the *mxd* genes under planktonic conditions (Figure 7, left) while it activates *mxd* expression in the biofilm (Figure 7, right).

The two different modes of action under planktonic and biofilm conditions could be explained as a consequence of additional *mxd* regulation at the transcriptional level. Unidentified transcriptional regulators could alter the transcriptional *mxd* output we observe in ∆*arcS* and ∆*arcA* mutants under planktonic and biofilm conditions. Due to the ecological differences that cells experience in planktonic culture and in a biofilm, the response in terms of *mxd* expression would then be very different. A further possibility is that ArcA receives signal inputs from other sensor kinases in addition to ArcS. Lassak *et al*. provided biochemical evidence showing that the ArcS/ArcA TCS in *S. oneidensis* MR-1 is only functional in the presence of a phosphotransfer domain HptA [14]. The function of phosphotransfer domains is not entirely clear, but they are thought to serve as a means to integrate signal inputs from several sensor kinases and relay that information to the cognate response regulator. Depending on whether a cell experiences planktonic growth conditions or is part of a structured biofilm, the input signals can vary greatly, and, as a consequence, *mxd* expression can be very different in these environments. Further investigation will be

 necessary to determine the differences in *mxd* regulation by ArcS/ArcA under planktonic conditions and in a biofilm. Additionally, based on the provided evidence we cannot entirely exclude that ArcS/ArcA regulation of the *mxd* operon is indirect. Biochemical analysis will have to be performed to show direct interaction of ArcA with the *mxd* promoter.

The signal input for the ArcS sensor kinase in *S. oneidensis* MR-1 has not yet been identified. The sensor kinase ArcB in *E. coli* responds to changes in oxygen

 availability by sensing the redox state of the quinone pool. Based on the homology of the two Arc systems, it is possible that Arc has a similar function in *S. oneidensis* MR-1. To test whether expression of the *mxd* operon was regulated in response to metabolic changes, and more specifically to redox changes (oxic/anoxic), via the Arc system, experiments with *S. oneidensis* MR-1 wild type strains carrying a copy of *lacZ* fused to the *mxd* promoter under controlled chemostat-like conditions had been conducted. Strains were cultivated in a batch fermenter in LB medium or LB medium amended with 50 mM sodium fumarate and grown aerobically (dissolved oxygen was monitored during the entire experiment) to exponential phase and then shifted to anoxic growth conditions by depleting oxygen. *β*-galactosidase activity in these strains was monitored before and up to 12 hours after the shift. No change in *mxd* expression was observed upon oxygen depletion (data not shown). This led us to the conclusion that a change in redox conditions and metabolic activity per se (induced by electron acceptor starvation) did not play a role in Arc mediated *mxd* regulation. Based on recently published data, revealing that *Shewanella* ArcS possesses additional sensory regions when compared to ArcB in *E. coli*, the Arc system in *Shewanella* species might also be able to sense other unknown environmental signals [28].

## Conclusions

The presented data show that carbon starvation is the dominant environmental cue triggering *mxd* induction in *S. oneidensis* MR-1*,* and that the *mxd* genes are controlled transcriptionally by ArcS/ArcA and BarA/UvrY. Interestingly, BarA/UvrY appears to be a major regulator of the *mxd* genes and is primarily responsible for induction in cells that have entered stationary phase and are exposed to starvation conditions while ArcS/ArcA appears to control *mxd* expression independent of growth phase. Although the signal for the BarA sensor histidine kinase has not been identified in *S. oneidensis* MR-1, it is reasonable to speculate that it is of similar molecular nature as the recently identified metabolites for *E. coli* BarA. However, considering that *E. coli* and *S. oneidensis* MR-1 inhabit different ecological niches, it is also conceivable that the signal input might be different. Thus, we hypothesize that based on our data carbon starvation could be the physiological signal sensed by BarA directly or indirectly. Both ∆*barA* and ∆*uvrY* mutants were unable to induce *mxd* expression when cells entered stationary phase. In fact, *mxd* expression in both mutants resembles the expression level observed in logarithmically growing wild type cells, indicating a possible role for BarA/UvrY in starvation response.

## Methods

### Strains and media

Strains used in this study are listed in Table 1. *E. coli* strains were grown at 37°C in lysogeny broth (LB) medium. Where necessary medium was solidified by 1.5% (w/v) agar and supplemented with 50 μg/mL kanamycin or 100 μg/mL ampicillin. *S. oneidensis* MR-1 strains were grown at 30°C in LB medium, lactate medium (LM) [0.02% (w/v) yeast extract, 0.01% (w/v) peptone, 10 mM (wt/vol) HEPES (pH 7.4), 10 mM NaHCO_3_ ] with a sodium lactate concentration of 50 mM or in minimal medium (MM) [1.27 mM K_2_ HPO_4_, 0.73 mM KH_2_PO_4_, 5 mM sodium 4-(2- hydroxyethyl)-1-piperazine-ethane-sulphonic acid (HEPES), 150 mM NaCl, 485 mM CaCl_2_, 9 mM (NH_4_)_2_SO_4_, 5 mM CoCl_2_, 0.2 mM CuSO_4_, 57 mM HBO, 5.4 mM FeCl, 1.0 mM MgSO_4_, 1.3 mM MnSO_4_, 67.2 mM Na_2_ EDTA, 3.9 mM Na_2_MoO_4_, 1.5 mM Na_2_SeO_4_, 2 mM NaHCO_3_, 5 mM NiCl_2_ and 1 mM ZnSO_4_, pH 7.4] amended with 50 mM sodium lactate as electron donor. Where necessary medium was solidified by 1.5% (w/v) agar and supplemented with 25 μg/mL kanamycin, 10 μg/mL tetracycline, 10 μg/mL gentamycine and 60 μg/mL 5-bromo-4-chloro-3-indolyl-beta- D-galactopyranoside (X-gal). Biofilms of *S. oneidensis* MR-1 were grown in LM amended with 0.5 mM sodium lactate (pH 7.4) or MM amended with 1.5 mM sodium lactate (pH 7.4). Where necessary medium was supplemented with 12.5 μg/mL kanamycin.

### Construction of *mxd* transcriptional reporter strains

*S. oneidensis* MR-1 *mxd* reporter strains were constructed by transcriptionally fusing various-length fragments of the *mxd* upstream region to *lacZ* and *gfp*. A promoterless copy of either *lacZ* or *gfp* in the appropriate vector served as a control.

### *LacZ* -reporter strains

To obtain a strain reporting on the transcriptional activity of *mxd*, a 450 bp fragment upstream of the *mxdA* translation initiation site was amplified with primers P*mxd*-fw-SphI and P*mxd*-rv-XbaI (Table 2) using *S. oneidensis* MR-1 genomic DNA as template. The *lacZ* gene was amplified from *E. coli* MG1655 genomic DNA using primers *LacZ*-fw-XbaI and *LacZ*-rv-PstI (Table 2). Subsequently, the two PCR products were purified from an agarose gel, restriction digested with *XbaI* and ligated. The fusion product was PCR amplified with primers P*mxd*-fw-SphI and LacZ-rv-PstI (Table 2), purified from an agarose gel, restriction digested with *XbaI* and *PstI* and ligated into vector pME6031 (pJM1). Truncations of the *mxd* promoter region were generated by amplification from pJM1 with the following primer combinations and subsequent ligation into pME6031 as described above:

150 bp upstream region: P*mxd*-fw-150-SphI and LacZ-rv-PstI.

250 bp upstream region: P*mxd*-fw-250-SphI and LacZ-rv-PstI.

300 bp upstream region: P*mxd*-fw-300-SphI and LacZ-rv-PstI.

#### Gfp -reporter strains

To construct a strain reporting on the transcriptional activity of *mxd*, a 450 bp fragment upstream of the *mxdA* translation initiation site was amplified with primers P*mxd*-fw-HindIII and P*mxd*-rv-XbaI (Table 2) using *S. oneidensis* MR-1 genomic DNA as template. The PCR product was purified from an agarose gel, restriction digested with *HindIII* and *XbaI* and ligated into a *HindIII* and *XbaI* restriction digested pProbe NT vector yielding pJM6. All reporter constructs were introduced into *E. coli* S17-λ pir by standard procedures. Plasmid was then prepared from positive clones and introduced into *S. oneidensis* MR-1 wild type or mutant strains by electroporation.

### Quantitative cell aggregation assay

*S. oneidensis* MR-1 wild type and mutant cells were grown in test tubes on a roller drum to exponential (OD_600_ = 0.3) and stationary phase (OD_600_ = 2.0) in minimal medium amended with 50 mM sodium lactate. Immediately after removing test tubes from the roller drum, one milliliter samples were taken and OD_600_ was determined. Further samples were taken after 15 minutes and 30 minutes. After measuring the optical density, cells were vigorously vortexed for 20 seconds and the optical density measurement was repeated. The ratio of OD_600_ before and OD_600_ after dispersion was calculated and used as an approximation to estimate the extend of cell aggregation in the different strains.

### Construction of gene deletions

*S. oneidensis* MR-1 in-frame deletions were constructed by homologous recombination. The deletion constructs were created by amplifying the regions flanking the target gene. The fragment length was optimized to about 750 bp. The primers for the 5’- end fragment were 5-O (outside) and 5-I (inside) and the primers for the 3’- end fragment were 3-I (inside) and 3-O (outside). Subsequent to amplification, the flanking regions were fused via a complementary tag that was added to the 5’- end of each inner primer. The fusion product was inserted into the cloning vector pDS3.1 and the mobilizing strain *E. coli* S17-λ pir [38] was transformed with this sucicide vector. Functionality of the *sacB* gene was verified before transferring the deletion vector by conjugation into the *S. oneidensis* MR-1 target strain. Single crossover events were selected for on LB plates containing gentamycine and confirmed by using two primer combinations: 1) primer X-F and primer 3-O and 2) primer X-R and primer 5-O, whereas primer X-F and primer X-R will bind upstream and downstream of the flanking regions, respectively. The functionality of the *sacB* gene was verified in *S. oneidensis* MR-1 strains that tested positive for a single crossover event. Resolution of the integrated vector by a second crossover event was performed with a positive strain. This strain was grown in LB medium without selection and plated onto solid LB medium containing 10% sucrose. Deletion events were verified by PCR using primer X-F and primer X-R, where a successful deletion resulted in a PCR product with a size of the wild type product minus the size of the target gene.

### Construction of strains constitutively expressing GFP

Construction of *S. oneidensis* MR-1 strains constitutively expressing GFP was carried out using a Tn7 based delivery system [39]. GFP-labeling was performed by biparental mating. Cultures of *S. oneidensis* MR-1, AS262 and AS392 were grown in LB broth overnight. 0.5 mL of each culture containing about 10^8^ cells was washed twice in one culture volume of phosphate buffered saline (PBS). *S. oneidensis* MR-1 and AS262 cells were combined and resuspended in 250 μL PBS. AS392 cells were resupended in 250 μL PBS. 50 μL of the mixed *S. oneidensis* MR-1/AS262 cell suspension was combined with 50 μL AS392 cell suspension and spotted onto dry solidified LB medium. Petri dishes were incubated upright for 8 h at 30°C. The cell mass was then resuspended in PBS and spread onto LB agar supplemented with 10 μg/mL gentamycine to select for *S. oneidensis* MR-1 carrying a chromosomal insertion of the *gfp*-carrying Tn7. PCR was used to map the site of insertion in the *S. oneidensis* MR-1 genome.

### Tn5 mutagenesis and screen for *mxd* -deregulated mutants

Transposon mutagenesis was performed by mating AS536 with the donor strain *E. coli* BW20767 (AS259) harbouring suicide plasmid pRL27, which carries a hyperactive transposase and a Tn5-mini transposon with a kanamycin resistance cassette and a R6K origin of replication [40]. The mating was performed at a 1:1 donor-recipient ratio at room temperature for 6 h. Transconjugants were plated onto solid LB medium containing kanamycin, tetracycline and X-gal to qualitatively screen for deregulated *mxd* mutants. Mutants were identified based on the intenstity of their blue colony color compared to the non-mutagenized control strain AS536. The mutant phenotypes were quantitatively confirmed by *β* -galactosidase assay in liquid culture. The location of a Tn5 insertion was mapped by arbitrary primed PCR [4]. Chromosomal DNA was prepared from the mutants and two rounds of amplification were used to specifically amplify and enrich for the DNA flanking the insertion site. In the first round primer tpnRL 17-1-O or tpnRL 13-2-O, which are unique to one end of the transposon, and two different arbitrary primers ARB1 and ARB6 [4] were used for amplification. Among the many possible amplified regions from the first round of PCR were products primed from the transposon and flanking chromosomal DNA. Products flanking the transposon were specifically amplified in the second round of PCR with primers tpnRL17-1 or tpnRL13-2 [4] and ARB2. After the second round of PCR the obtained PCR products were purified and subsequently subjected to DNA sequence analysis using primers tpnRL17-1 or tpnRL13-2. To identify the location of the transposon insertion, the resulting nucleotide sequences were compared with the *S. oneidensis* MR-1 sequence database by BLAST search:

(http://blast.ncbi.nlm.nih.gov).

### *β* -galactosidase assay

For *β* -galactosidase assays, *S. oneidensis* MR-1 strains were grown at 30°C in 250 mL flasks containing 25 mL of either LB medium or LM or 4M medium amended with 50 mM lactate. Specific activities were determined by a modified Miller method [41]. Briefly, cells were harvested during different growth stages and resuspended in Z-Buffer to an OD_600_ of 0.5-0.7. Samples were prepared in triplicates by adding 100 μL of cell suspension to 900 μL Z-buffer with 0.27% (v/v) β -mercaptoethanol, 50 μL chloroform and 100 μL 0.1% SDS and vortexing for 10 seconds. After equilibration at 28°C for 10 minutes, the reaction was started by addition of 0.2 mL o-nitrophenyl-D-galactoside (ONPG) [4 mg * mL^-1^ ] and incubating the samples at 28°C. The reactions were stopped with 0.5 mL Na_2_ CO_3_ [1M] when samples developed a yellowish color. Samples were centrifuged for 5 minutes at 13,000 rpm and OD_420_ was recorded. Specific activities were expressed as Miller Units and calculated as follows:

1 Miller Unit = 1000 * (OD_420_ )/(t * V * OD_600_ ),

where t = time

V= volume

OD= optical density

### Biofilm cultivation

Biofilms were grown at 30°C in three-channel flow cells as decribed previously [12]. Briefly, LB overnight cultures of the relevant *S. oneidensis* MR-1 strains were diluted 1/100 in LB and grown to early stationary phase. Then the optical density at 600 nm was adjusted to 0.01 in 4M MM or LM without carbon source. 1 mL of the OD_600_ = 0.01 cell suspension was injected into each flow channel while the medium flow was stopped. The flow cells were inverted (glass slide facing bottom) and incubated for 40 min at 30°C. After incubation flow cells were reverted and medium was pumped through the flow cell at a constant velocity of 0.3 mm/s per channel by a Watson-Marlow Bredel (Cornwall, United Kingdom) 205S peristaltic pump. Biofilm studies were carried out in triplicate in at least two independent experiments.

### Biofilm image acquisition and processing

Microscopic visualization of biofilms was performed using an upright Leica TCS SP2 AOBS confocal laser scanning microscope (CLSM; Leica Microsystems, Wetzlar, Germany) using the following objectives: HCX PL APO 63X/1.2 W CORR CS and HC PL FLUORTAR 20X/0.5. For three-dimensional reconstruction of biofilm images, CLSM images were processed with the IMARIS software package (Bitplane AG, Zuerich, Switzerland) and Adobe Photoshop.

### Flow cytometry

24 h old LM grown biofilm of *S. oneidensis* MR-1 wild type and mutant cells carrying a P_*mxd*_::*gfp* reporter construct were harvested from the flow chamber, passed 50 times through a 25 gauge needle to suspend any cell aggregates and fixed in 2% paraformaldehyde. Flow cytometry data were obtained using a BD FACSCalibur flow cytometer (BD Biosciences, San Jose, CA). Samples were analysed using the 488 nm excitation from an argon-ion LASER at 15 mW. Detector voltages were set at defined values [800 V for the fluorescence channel (FL1) and both the FL1 and forward scatter channel amp gain were set to logarithmic scale] prior to the experimental analysis in which samples were run in succession on the same day. A control sample of wild type strain MR-1 biofilm cells carrying promoterless *gfp* (AS838) was used for background subtraction. Data acquisition and analysis was performed with CellQuest (BD Biosciences) software.

## Competing interests

The authors declare that they have no competing interests.

## Authors’ contributions

JM carried out the majority of the experimental work. SS constructed the *mxd*::*lacZ* reporter plasmid and KAS participated in the transposon mutagenesis. JM and AMS conceived the experiments and drafted the manuscript. All authors read and approved the final manuscript.

## Supplementary Material

Additional file 1: Figure S1 Expression of *mxd* in *S. oneidensis* MR-1 wild type and ∆*arcS* and ∆*arcA* mutant biofilms. GFP fluorescence intensities of *S. oneidensis* MR-1 wild type, ∆*arcS* and ∆*arcA* biofilm mutant cells measured by flow cytometry. All strains carried a P_*mxd*_::*gfp* reporter and were grown in LM in a hydrodynamic flow chamber for 24 h. Biofilm cells of wild type strain MR-1 carrying promoterless *gfp* were used as a control for background subtraction. Fluorescence intensities were calculated as a percentage of the total cell population after background subtraction. Data represent one of two performed experiments with similar trends. Click here for file
