# Supplementary figures and images for: The mxd operon in Shewanella oneidensis MR-1 is induced in response to starvation and regulated by ArcS/ArcA and BarA/UvrY
Source: BMC Microbiol. 2013 May 27;13:119. doi: 10.1186/1471-2180-13-119 (PMC3691769; doi:10.1186/1471-2180-13-119)

## Slide 1
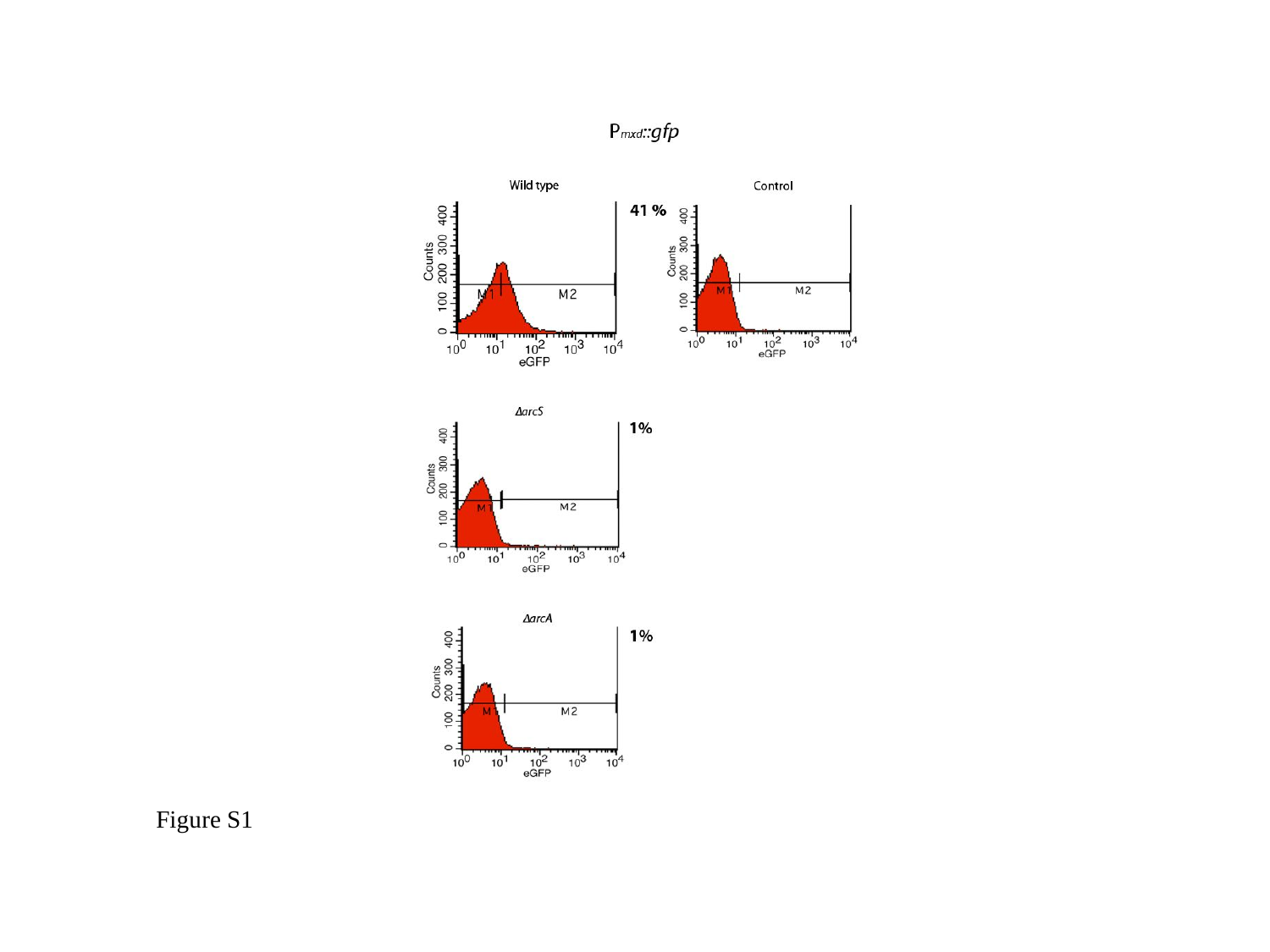

Figure S1

Supplement: Additional file 1: Figure S1 — Expression of mxd in S. oneidensis MR-1 wild type and ∆arcS and ∆arcA mutant biofilms. GFP fluorescence intensities of S. oneidensis MR-1 wild type, ∆arcS and ∆arcA biofilm mutant cells measured by flow cytometry. All strains carried a Pmxd::gfp reporter and were grown in LM in a hydrodynamic flow chamber for 24 h. Biofilm cells of wild type strain MR-1 carrying promoterless gfp were used as a control for background subtraction. Fluorescence intensities were calculated as a percentage of the total cell population after background subtraction. Data represent one of two performed experiments with similar trends. [file 1471-2180-13-119-S1.pptx]
